# Beyond pregnancy – the neglected burden of mortality in young women of reproductive age in Bangladesh: a prospective cohort study

**DOI:** 10.1111/1471-0528.12245

**Published:** 2013-05-07

**Authors:** AB Labrique, SS Sikder, L Wu, M Rashid, H Ali, B Ullah, AA Shamim, S Mehra, R Klemm, H Banu, KP West, P Christian

**Affiliations:** a Department of International Health, Johns Hopkins Bloomberg School of Public HealthBaltimore, MD, USA; b The JiVitA Maternal and Child Health Research ProjectPoschim Para, Gaibandha, Bangladesh; c Maternal and Neonatal Health Initiative, United Nations Population FundAladatpur, Narail, Bangladesh; d Department of Gynecology, Institute of Child and Mother HealthDhaka, Matuail, Bangladesh

**Keywords:** Bangladesh, low-income settings, non-communicable disease, reproductive age, South Asia, verbal autopsy

## Abstract

**Objective** To describe proportionate mortality and causes of death unrelated to pregnancy.

**Design** Prospective cohort study.

**Setting** Rural northwest Bangladesh.

**Population** A cohort of 133 617 married women of reproductive age.

**Methods** Verbal autopsies were conducted for women who died whilst under surveillance in the cohort trial. Physician-assigned causes of death based on verbal autopsies were used to categorise deaths.

**Main outcome measures** The proportion of deaths due to non-communicable diseases, infectious diseases, injury or pregnancy.

**Results** Of the 1107 deaths occurring among women between 2001 and 2007, 48% were attributed to non-communicable diseases, 22% to pregnancy, 17% to infections, 9% to injury and 4% to other causes.

**Conclusions** Although focus on pregnancy-related mortality remains important, more attention is warranted on non-communicable diseases among women of reproductive age.

## Introduction

Despite the high proportion of adult female mortality attributed to non-communicable diseases (NCDs), most research on women of reproductive age remains focused on death caused by pregnancy and childbirth. According to the 2010 Bangladesh Maternal Mortality and Health Care Survey, cancer was identified as the leading cause of death among women of reproductive age in Bangladesh, accounting for 21% of deaths between 2001 and 2009. Deaths from circulatory diseases (16%) and maternal deaths (14%) followed close behind.^1^ Although the 2011 UN Summit on Non-Communicable Diseases called for greater attention to NCDs, population-based data on mortality resulting from NCDs among women of reproductive age are lacking. Using data from a longitudinal, 6-year cohort study following more than 130 000 women of reproductive age in rural Bangladesh, we sought to explore proportionate mortality attributed to pregnancy and non-pregnancy-related causes and to describe the proportionate causes of death unrelated to pregnancy in this relatively young cohort. This report presents some of the only population-based, prospective data on cause-specific mortality in this important South Asian demographic.

## Methods

We used data from a surveillance system designed to identify pregnant women for a nutritional intervention trial, called JiVitA-1, conducted between 2001 and 2007.^2^ Approximately 133 617 married women of reproductive age (14–45 years) were under surveillance for pregnancy in a population of 650 000 living in northwest rural Bangladesh.^2^ The study site, a contiguous area of 435 km^2^, was selected for its population density (approximately 1000 people/km^2^) and rural, agrarian characteristics that reflected the 35th percentile for most public health indicators in rural Bangladesh.^3^ The trial assessed the efficacy of antenatal to postnatal weekly supplementation of women with vitamin A or β-carotene in reducing mortality related to pregnancy, fetal loss and infant mortality. The detailed methods and findings of this trial, including causes of pregnancy-related mortality, have been published elsewhere.^2^–^3^

Every 5 weeks, field workers visited married women of reproductive age who were eligible to become pregnant to ascertain pregnancies and to prospectively capture deaths. Women who became pregnant were enrolled and followed weekly for supplementation and recording of pregnancy outcomes. Verbal autopsy interviews were conducted for all women who died whilst under surveillance, regardless of pregnancy status. After a woman had been reported as dead, a physician interviewer visited the home of the deceased to ask the spouse, other relatives or neighbours to describe the circumstances surrounding the woman’s death as part of a detailed verbal autopsy interview. When available, medical records of the deceased woman were also reviewed. The verbal autopsy tools, locally adapted from standard instruments developed by the World Health Organization (WHO), included structured morbidity modules and an open-ended narrative of the events and healthcare seeking leading up to the woman’s death.^4^ Physician interviewers were general physicians trained in rapport building and probing techniques. Interviews typically took between 60 and 90 minutes to complete. A team of two physicians, consisting of an obstetrician and a specialist in epidemiology, reviewed verbal autopsies to assign a proximal, or dominant, cause of death and up to five underlying or contributing causes of death for each woman. A proximal cause of death was assigned by two physician reviewers independently, followed by a discussion to assign a consensus cause of death.

Using the *International Classification of Diseases*, 10th Revision (ICD-10) classification, a pregnancy-related death was defined as the death of a woman whilst pregnant or within 42 days of termination of the pregnancy, irrespective of the cause of death. A non-pregnancy-related death was defined as the death of a woman occurring outside the time period of pregnancy through 42 days following pregnancy termination. The timing of death in relation to pregnancy (if applicable) was assigned based on study records. We counted the total number of deaths, pregnancy related or otherwise, and calculated proportionate mortality by cause of death. Physician-assigned proximal causes of death determined from consensus were used to classify the causes of death into four major categories, (1) NCD (including circulatory diseases, cancer, gastrointestinal diseases, diabetes, renal disorders, asthma, epilepsy and rheumatic disorders); (2) infectious disease; (3) injury; and (4) pregnancy, based on WHO’s 2007 Verbal Autopsy Standards.^4^

## Results

We recorded deaths that occurred between August 2001 and August 2007, a 6-year period during which continuous 5-weekly population surveillance and assessment of vital events were conducted. In this time frame, we recorded a total of 1179 deaths among enrolled women. Seventy-two (6%) verbal autopsies could not be completed as the families had moved or were not met after three attempts. Of the 1107 deaths among women of reproductive age for which verbal autopsies were completed, 48% (*n* = 530) were attributed to NCDs, 22% (*n* = 238) to pregnancy, 17% (*n* = 185) to infectious causes, 9% (*n* = 101) to injuries and 4% (*n* = 49) to other causes (Table 1).

**Table 1:** Causes of death among women of reproductive age for whom verbal autopsies were completed (*n* = 1107) between August 2001 and August 2007 in northwest rural Bangladesh

Cause of death	Number of deaths	Percentage of total deaths (*n* = 1107)
**Non-communicable disease category**	**530**	**47.9**
Circulatory disease (Verbal Autopsy Code-04)^4^	212	19.2
*Cerebrovascular accident/stroke*	*104*	*9.4*
*Heart failure*	*68*	*6.1*
*Heart attack/ischaemic heart disease*	*32*	*2.9*
*Heart disease*	*7*	*0.6*
*Pericardial effusion*	*1*	*0.1*
Cancer (VA-02)	168	15.2
Gastrointestinal disorders (VA-06)	94	8.5
Diabetes (VA-03.03)	25	2.3
Renal disorders (VA-07)	20	1.8
Asthma (VA-05.02)	5	0.5
Epilepsy (VA-08.02)	4	0.4
Rheumatic disorders (VA-08)	3	0.3
**Infectious category** (Verbal Autopsy Code-01)^4^	**185**	**16.7**
Diarrhoea/dehydration (VA-01.01)	85	7.7
Tuberculosis (VA-01.03)	56	5.1
Malaria (VA-01.10)	12	1.1
Sepsis (VA-01.99)	10	0.9
Enteric fever/typhoid fever (VA-01.02)	5	0.5
Tetanus (VA-01.03)	5	0.5
Meningitis (VA-01.11)	5	0.5
Other (diphtheria, rabies, tonsillitis)	5	0.5
Intestinal infectious disease (VA-01.01)	1	0.1
Respiratory infection (VA-01.13)	1	0.1
**Injury category** (Verbal Autopsy Code-11)^4^	**101**	**9.1**
Intentional injuries	73	6.6
*Suicide* (VA-11.10)	*63*	*5.7*
*Homicide* (VA-11.11)	*10*	*0.9*
Unintentional injuries (VA-11.01 to 11.09)	28	2.5
**Pregnancy-related deaths**	**238**	**21.5**
**Other**	**49**	**4**.**4**
Anaemia (VA-03.01)	39	3.5
Anaphylactic shock	3	0.3
Malnutrition (VA-03)	3	0.3
Pulmonary embolism	2	0.2
Haemorrhage	1	0.1
Anorexia nervosa	1	0.1
**Uncertain** (VA-99)^4^	**4**	**0.4**
**Total**	**1107**	**100.0**

Among NCD deaths, circulatory diseases accounted for the largest percentage of deaths (40%, *n* = 212), with cerebrovascular accident as the leading cause of circulatory disease death (*n* = 104). Cancer accounted for 15% (*n* = 168) of all deaths, with gastric cancer as the top cause (*n* = 38), followed by liver cancer (*n* = 19) and cervical cancer (*n* = 8). Among the 185 recorded deaths resulting from infectious causes, diarrhoea with dehydration was listed as the top cause (8% of total deaths; *n* = 85), followed by tuberculosis (5% of total deaths, *n* = 56) and sepsis (1% of total deaths, *n* = 10). Of the 101 deaths (9%) caused by injury, 73% (*n* = 73) were classified as intentional, defined as injuries that were self-inflicted or inflicted by another person (Table 1). Twenty-seven percent (*n* = 28) of injury deaths, categorised as unintentional, were caused by road traffic accidents, drowning, electric shock and burns. For four women, the cause of death remained uncertain as the verbal autopsies did not provide sufficient information.

## Discussion

### Main findings

In this study population of rural Bangladesh, characterised by an average total fertility rate of 2.3 births per woman, contraceptive prevalence rate of 61% and an unmet need for family planning of 12%,^5^ NCDs appeared to be the leading cause of death among women of reproductive age, followed by pregnancy, infectious disease and injury. Circulatory diseases accounted for the largest proportion of NCD deaths, followed by cancer.

### Interpretation

Our study suggests that NCDs play a prominent role in female mortality in rural Bangladesh. In the South-East Asia region, WHO estimates that 48% of deaths among adult females are caused by NCDs, followed by 21% by injury, 16% by infectious diseases and 13% as a result of pregnancy.^6^ This research suggests a large proportion of NCD mortality caused by circulatory diseases. Other authors have noted a genetic susceptibility of South Asians to circulatory disease, with Bhopal et al. suggesting potential genetic differences between fetal and early life metabolism and metabolism in middle age as potential risk factors.^10^ Population-based studies in South Asia suggest high rates of risk factors for circulatory disease, such as high blood pressure, high cholesterol levels, low high-density lipoprotein (HDL) cholesterol, insulin resistance and diabetes, as well as lifestyle factors, such as poor diet, tobacco consumption and a sedentary lifestyle.^8^–^9^ These risk factors may contribute to the large proportion of deaths attributable to circulatory disease in this relatively young female cohort.

This study demonstrates the potential contribution of verbal autopsy data to the ascertainment of population-based mortality, as indicated by Jha et al. in their studies using verbal autopsies for the identification of causes of death in settings throughout India.^10^ As the women in this study population have similar socioeconomic and demographic characteristics to rural women throughout Bangladesh, these findings may be a reasonable reflection of proportionate mortality in women in similar populations across the greater Gangetic floodplain. Following a cohort of 43 510 women of reproductive age in the southeastern plains of Nepal between 1994 and 1997, West found that over two-thirds of deaths were unrelated to pregnancy (K. P. West Jr, pers. comm. 2012).

In addition, access to health care in the study area of Gaibandha was similar to the distribution of public facilities throughout rural Bangladesh. Within the study district of Gaibandha, there was one public district hospital, one maternal and child welfare clinic, two subdistrict health facilities and five private clinics. The district to the northwest, Rangpur District, included a tertiary medical college hospital, a maternal and child welfare clinic, and over 80 private clinics. Because the allocation of public facilities is centrally determined by the Ministry of Health and Family Welfare based on administrative divisions, the distribution of health facilities in the study districts is similar to that in other regions of the country.^11^

### Strengths and limitations

With the use of a comprehensive surveillance system, this population-based survey was able to prospectively detect mortality with a low chance of missing deaths. Moreover, the completion of meticulous and thorough verbal autopsies allowed for the assignment of specific proximal causes of death for the majority of cases. To reduce bias, physician interviewers and reviewers were trained to ensure the standardised collection of verbal autopsy data, as well as standardised procedures to assign causes of death.

As these verbal autopsy instruments were designed to capture maternal deaths, their validity for the ascertainment of non-maternal deaths is unknown. Studies conducted by Kumar et al. from India suggest that the validity of verbal autopsies to discern circulatory diseases was lower than for other adult causes of death.^10^ Thus, there may have been possible misclassification of deaths caused by circulatory disease. The verbal review process to assign causes of death, in which physicians discussed the consensus cause of death, was applied to minimise potential misclassification.

Verbal autopsies were carefully reviewed by physicians to assign dominant causes of death with the use of protocols developed by WHO for settings lacking vital registration.^4^ Because this analysis focused on dominant causes of death, the contribution of underlying causes of death, such as malnutrition, was not estimated.

This analysis was based on data from a cohort trial which sought to test the effect of antenatal micronutrient supplementation on pregnancy-related mortality. Women who became pregnant whilst under surveillance were followed up closely for detailed information on background, age and other data. For women who did not become pregnant, however, data on age at death was largely unavailable.

## Conclusions

In the light of the 2011 UN Summit Non-Communicable Diseases and the UN Secretary General’s Report on NCDs calling for further research attention to NCDs in low-income settings, this study will help to fill an important data gap on mortality attributable to NCDs in an important demographic. Further research on diet and lifestyle factors that contribute to the risk for NCDs in these contexts may inform health promotion and behaviour modification activities. For decades, global attention has been focused on reducing maternal mortality, given the greater visibility and enumeration of women throughout pregnancy and childbirth. Although continued focus on pregnancy-related mortality remains important, attention is warranted on the substantial burden of mortality unrelated to pregnancy among women of reproductive age. Comprehensive and prophylactic interventions that focus on improving nutrition, health education and early screening for chronic disease may be needed to address preventable deaths in these young women. These data should help to stimulate further research and investment in programme interventions to address NCDs, alongside efforts to make pregnancy safer.

### Disclosure of interests

None of the authors listed above have any known conflict of interest.

### Contribution to authorship

This analysis represents the work of a large, multi-country team which managed the JiVitA-1 prospective cohort trial. ABL, co-investigator, had primary responsibility for the conception of the analysis, oversight of the implementation of the research, analysis and preparation of the manuscript. SSS performed quantitative analyses of the dataset, drafted the manuscript and incorporated critical revisions. LW assisted with data acquisition, cleaning and analysis. MR, as a co-investigator, oversaw the verbal autopsy review and data acquisition process. HA and BU were responsible for the management of data collection, training, oversight and interpretation of the data. AAS, co-investigator, assisted with study implementation, and HB assisted with data acquisition. SM, RK, KPW and PC were responsible for study design, interpretation of the data and critical revisions of the manuscript. All authors provided critical revisions of the manuscript and final approval of its content.

### Details of ethics approval

This analysis utilised data from the JiVitA-1 trial (Clintrials.gov number NCT00198822). This study was approved by the Johns Hopkins Bloomberg School of Public Health Institutional Review Board and the Bangladesh Medical Research Council.

### Funding

Financial support was provided by the Bill and Melinda Gates Foundation, Seattle, WA, USA (Global Control of Micronutrient Deficiency, Grant 614), Sight and Life Research Institute (Baltimore, MD, USA), with original trial support also from the US Agency for International Development, Washington DC (GHS-A-00-03-00019-00). Partial support for this data analysis was provided by the Johns Hopkins Bloomberg School of Public Health through a Global Field Experience Fund (Johns Hopkins Office of External Affairs), a Framework Award in Global Health (Johns Hopkins Center for Global Health) and a Delta Omega Scholarship (Johns Hopkins Delta Omega Honor Society).

## References

[1] National Institute of Population Research and Training (NIPORT), MEASURE Evaluation, and ICDDR,B (2012). Bangladesh Maternal Mortality and Health Care Survey 2010.

[2] West KP, Christian P, Labrique AB, Rashid M, Shamim AA, Klemm RD (2011). Effects of vitamin A or beta carotene supplementation on pregnancy-related mortality and infant mortality in rural Bangladesh: a cluster randomized trial. J Am Med Assoc.

[3] Labrique AB, Christian P, Klemm RD, Rashid M, Shamim AA, Massie A (2011). A cluster-randomized, placebo-controlled, maternal vitamin A or beta-carotene supplementation trial in Bangladesh: design and methods. Trials.

[4] World Health Organization (2007). Applying ICD-10 to verbal autopsy. Verbal Autopsy Standards: Ascertaining and Attributing Causes of Death.

[5] Mehra S, National Institute of Population Research and Training, Mitra and Associates (2012). 2011 Bangladesh Demographic and Health Survey.

[6] World Health Organization (WHO) (2009). Women and Health: Today’s Evidence Tomorrow’s Agenda.

[7] Bhopal R (2002). Epidemic of cardiovascular disease in South Asians. BMJ.

[8] Singh RB, Ghosh S, Niaz AM, Gupta S, Bishnoi I, Sharma JP (1995). Epidemiologic study of diet and coronary risk factors in relation to central obesity and insulin levels in rural and urban populations of north India. Int J Cardiol.

[9] Gupta R, Prakash H, Gupta VP, Gupta KD (1997). Prevalence and determinants of coronary heart disease in a rural population of India. J Clin Epidemiol.

[10] Jha P, Gajalakshmi V, Gupta PC, Kumar R, Mony P, Dhingra N (2006). Prospective study of one million deaths in India: rationale, design, and validation results. PLoS Med.

[11] Management Information Systems Directorate General of Health Services (2012). Health Bulletin 2011.

[12] Kumar R, Thakur JS, Rao BT, Singh MM, Bhatia SP (2006). Validity of verbal autopsy in determining causes of adult deaths. Indian J Public Health.

